# A comparative study of the ultrastructural characteristics of the mature spermatozoa of two fellodistomids *Tergestia clonacantha* and *T. laticollis* and contribution to the phylogenetic knowledge of the Gymnophalloidea

**DOI:** 10.1051/parasite/2020066

**Published:** 2020-11-27

**Authors:** Papa Ibnou Ndiaye, Bernard Marchand, Cheikh Tidiane Bâ, Jean-Lou Justine, Rodney Alan Bray, Yann Quilichini

**Affiliations:** 1 Laboratory of Evolutionary Biology, Ecology and Management of Ecosystems, Faculty of Sciences and Techniques, Cheikh Anta Diop University of Dakar BP 5055 Dakar Senegal; 2 UMR 6134 SPE, CNRS – Università di Corsica, Campus Grimaldi 20250 Corte Corsica France; 3 Institut Systématique Évolution Biodiversité (ISYEB), Muséum National d’Histoire Naturelle, CNRS, Sorbonne Université, EPHE, Université des Antilles 57 rue Cuvier, CP 51 75231 Paris Cedex 05 France; 4 Department of Life Sciences, Natural History Museum Cromwell Road SW7 5BD London United Kingdom

**Keywords:** *Tergestia clonacantha*, *T. laticollis*, Ultrastructure, Spermatozoon, Gymnophalloidea, Fellodistomidae

## Abstract

The ultrastructure of the mature spermatozoa of *Tergestia clonacantha* and *T. laticollis* collected from the digestive tracts of fishes from New Caledonia is described using transmission electron microscopy and compared to that of related species. The spermatozoa of the two species exhibit the general pattern described in most digeneans, namely two axonemes with the 9 + “1” pattern of the Trepaxonemata, nucleus, mitochondrion, cortical microtubules, an external ornamentation of the plasma membrane, spine-like bodies and granules of glycogen. The spermatozoa of *T. clonacantha* and *T. laticollis* show the same ultrastructural model with some specificities in each case, particularly in the disposition of the structures in the posterior extremities of the spermatozoon. This study confirms that ultrastructural characters of the mature spermatozoon are useful tools for the phylogenetic analysis of the Digenea.

## Abstract (fr)

L’ultrastructure des spermatozoïdes mûrs de *Tergestia clonacantha* et T. *laticollis*, prélevés dans le tube digestif de poissons de Nouvelle-Calédonie, est décrite par microscopie électronique à transmission et comparée à celle d’espèces apparentées. Les spermatozoïdes des deux espèces présentent la structure générale décrite chez la plupart des digènes, à savoir deux axonèmes du type 9 + « 1 » des Trepaxonemata, un noyau, une mitochondrie, des microtubules corticaux, des ornementations externes de la membrane plasmique, des corps épineux et des granules de glycogène. Les spermatozoïdes de *T. clonacantha* et *T. laticollis* présentent le même modèle ultrastructural avec quelques spécificités dans chaque cas, notamment dans la disposition des structures aux extrémités postérieures du spermatozoïde. Cette étude confirme que les caractères ultrastructuraux du spermatozoïde mûrs sont des outils utiles pour l’analyse phylogénétique des Digenea.

## Introduction

It is now recognized that the ultrastructure of mature spermatozoa constitutes a useful complement to molecular, morphological and biological data in the understanding of the phylogeny of the Platyhelminthes. Studies of sperm structure in the poorly known orders and additional comparative studies are expected to provide new information for elucidation of phylogenetic relationships [2, 7, 11, 17, 25, 28]. Characters of special interest are: (1) presence or absence of mitochondria in the mature spermatozoon and their number, (2) the type of spermiogenesis, (3) presence or absence of the intercentriolar body during spermiogenesis, (4) the striated roots, (5) the periaxonemal sheath, (6) apical electron dense material in the spermatozoon, (7) the apical cone, (8) the ultrastructure of the anterior or posterior extremities, (9) the form and disposition of the nucleus, (10) the number and distribution of cortical microtubules, (11) the spine-like bodies, (12) the external ornamentation of the plasma membrane, (13) the disposition of the glycogen granules, and (14) the expansion of the plasma membrane.

The subclass Digenea is one of the largest groups of internal metazoan parasites with more than 18,000 recognized species belonging to 150 families [9]. Spermatological data are available for more than 100 species. The available data are not equally distributed among all the taxonomic levels of Digenea. Some families have never been studied, while some genera have already been the subject of several studies. Among these genera, we considered: the Aephnidiogenidae *Holorchis* [3, 12], the Bucephalidae *Prosorhynchus* [18, 26], the Dicrocoeliidae *Dicrocoelium* [1, 8], the Diplodiscidae *Diplodiscus* [5, 10], the Fasciolidae *Fasciola* [21, 22], the Hemiuridae *Lecithochirium* [23], the Lepocreadiidae *Bianium* [30], the Notocotylidae *Notocotylus* [20, 24], the Opecoelidae *Allopodocotyle* [6, 15], *Helicometra* [16, 29] and *Nicolla* [27, 28], the Opisthorchiidae *Opisthorchis* [19, 32], and the Zoogonidae *Lecithostaphylus* [4, 14].

In this study, we describe the ultrastructural features of the mature spermatozoa of *Tergestia clonacantha* and *T. laticollis*. Only the fellodistomid *Tergestia acanthocephala* [13] has been studied among the five families of Gymnophalloidea. The aim of this work was to add spermatological data for the Fellodistomidae and the Gymnophalloidea. Moreover, we aimed to confirm the stability of ultrastructural sperm characters at a low taxonomic level (species) to emphasize their usefulness to discriminate higher taxonomic groups (orders or superfamilies).

## Material and methods

Live adult specimens of *Tergestia clonacantha* Manter, 1963 and *T. laticollis* (Rudolphi, 1819) Stossich, 1899 were collected from the digestive tracts of *Hemirhamphus far* and *Carangoides chrysophrys*, respectively, off Nouméa (New Caledonia). Specimens were rinsed with 0.9% NaCl solution and fixed in cold (4 °C) 2.5% glutaraldehyde in 0.1M sodium cacodylate buffer at pH 7.2, postfixed in cold (4 °C) 1% osmium tetroxide in the same buffer for 1 h, dehydrated in ethanol and propylene oxide, embedded in Spurr’s resin and finally polymerized at 60 °C for 24 h. Ultrathin sections (60–90 nm thick) were cut on an ultramicrotome (Power tome PC, RMC Boeckeler) with diamond knife and placed on copper and gold grids. Copper grids were double stained with uranyl acetate and lead citrate. To reveal the presence of glycogen, golds grids were stained according to the method of Thiéry [31] with periodic acid (PA), thiocarbohydrazide (TCH) and silver proteinate (SP) as follows: 30 min in 10% PA, rinsed in Milli-Q water, 2 h in TCH, rinsed in acetic solution and Milli-Q, 30 min in 1% SP in the dark and rinsed in Milli-Q water. Then, copper and gold grids were examined using a Hitachi H-7650 electron microscope operated at 80 kV in the “Service d’Étude et de Recherche en Microscopie Électronique” of the University of Corsica (Corte, France).

## Results

The observation of numerous transverse sections in the seminal vesicle of the spermatozoa of *Tergestia clonacantha* and *T. laticollis* under the transmission electron microscope permitted us to distinguish four regions (I–IV) from the anterior to the posterior parts of mature spermatozoa of these species. The spermatozoa exhibit the main ultrastructural characteristics generally described in digeneans, namely two axonemes with the 9 + “1” pattern of the Trepaxonemata, nucleus, mitochondria, cortical microtubules, an external ornamentation of the plasma membrane, spine-like bodies, and granules of glycogen. However, the spermatozoa of *T. clonacantha* and *T. laticollis* present some peculiarities that we describe below.

**Figure 1:**
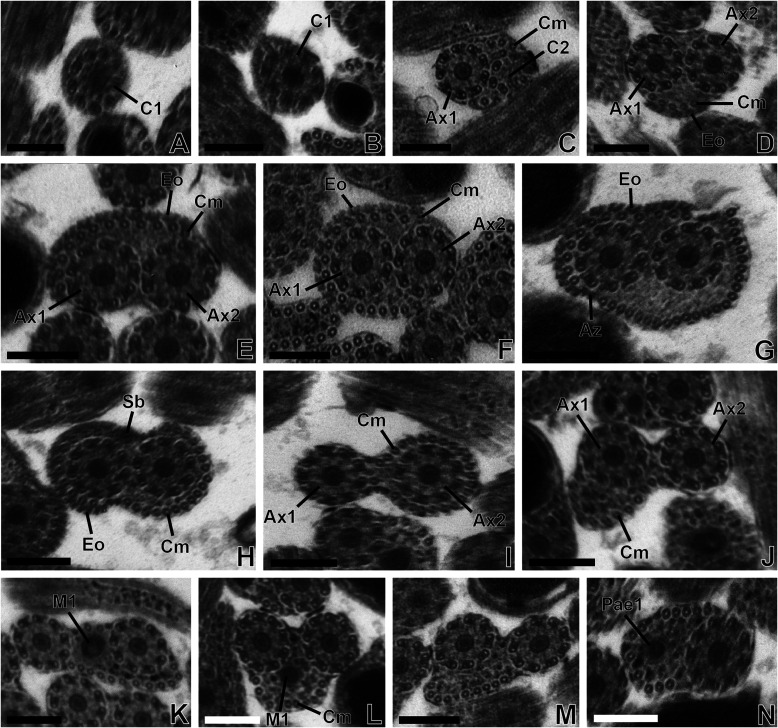
(A–N) Transmission electron micrographs of regions I, II and III of the mature spermatozoon of *Tergestia clonacantha.* Scale bars = 0.2 μm. (A–C) Cross-sections in the anterior extremity (or region I) of the spermatozoon. (A–B) Cross-sections in the anterior extremity of the spermatozoon with only the centriole (C1) of axoneme 1 (A × 1), cortical microtubules (Cm) and the centriole of axoneme 2 (C2). (C) Cross-section in the anterior extremity of the spermatozoon with axoneme 1, five cortical microtubules, and the centriole of axoneme 2 (C2). (D–H) Cross-sections in region II of the spermatozoon. (D) Cross-section in region II of the spermatozoon with the two axonemes (A × 1 and A × 2), cortical microtubules (Cm), and the external ornamentation of the plasma membrane (Eo). (D–H) Cross-sections in region II of the mature spermatozoon showing the two axonemes, an increasing number of cortical microtubules up to 36 in (G), and the external ornamentation of the plasma membrane and spine-like body (H). (I–N) Cross-sections in region III of the spermatozoon. (I) Cross-sections in region III of the mature spermatozoon showing only the two axonemes, a row of 23 cortical microtubules next to axoneme 2, a decrease of cortical microtubules up to 12 (J), the appearance of the first mitochondrion (M1) (K), a progressive decrease of cortical microtubules (L–M) and the disorganization of axoneme 1 (Pae1) (N).

*Region I* (Figs. 1A–1C, 3A–3F and 5I). This region corresponds to the anterior extremity of the mature spermatozoon characterized by the presence of a first axoneme in *T. clonacantha* (Fig. 1A) as in *T. laticollis* (Figs. 3A–3C). Thus, cortical microtubules and a second axoneme appear progressively (Figs. 1C and 3E–3F). There are only a few cortical microtubules in the anterior extremity of the spermatozoon. On the micrographs we counted 4 microtubules in *T. clonacantha* (Fig. 1C) and 6 in *T. laticollis* (Fig. 3F).

**Figure 2:**
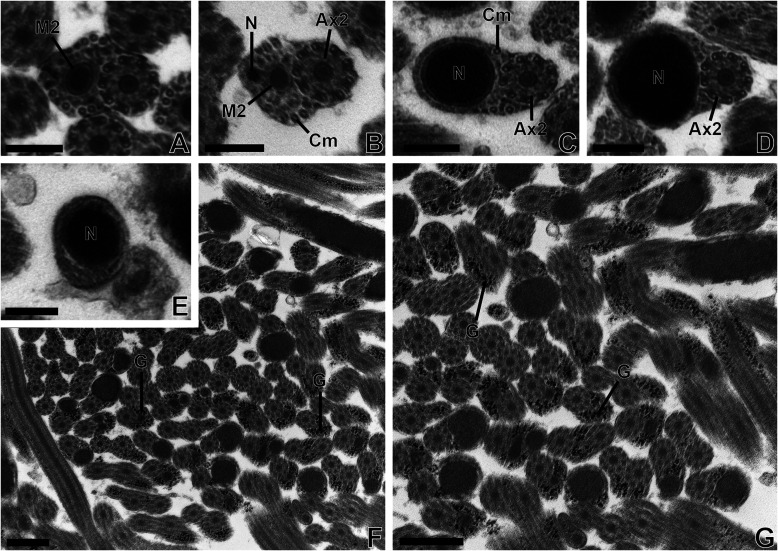
(A–E) Transmission electron micrographs of region IV (posterior region) of the mature spermatozoon of *Tergestia clonacantha.* Scale bars = 0.2 μm. Cross-sections in region IV of mature spermatozoon of *Tergestia clonacantha* showing the appearance of the second mitochondrion (M2) (A), then the nucleus (N) (B). (C) Cross-sections showing the disappearance of the first mitochondrion, a progressive disappearance of microtubules (C–D) and axoneme 2 (E). Then, the posterior extremity of the spermatozoon exhibiting only the nucleus (E). (F–G) Transmission electron micrographs showing many sections of the mature spermatozoon of *Tergestia clonacantha* with granules of glycogen (G) highlighted by the Thiéry method. Scale bars = 0.5 μm.

**Figure 3:**
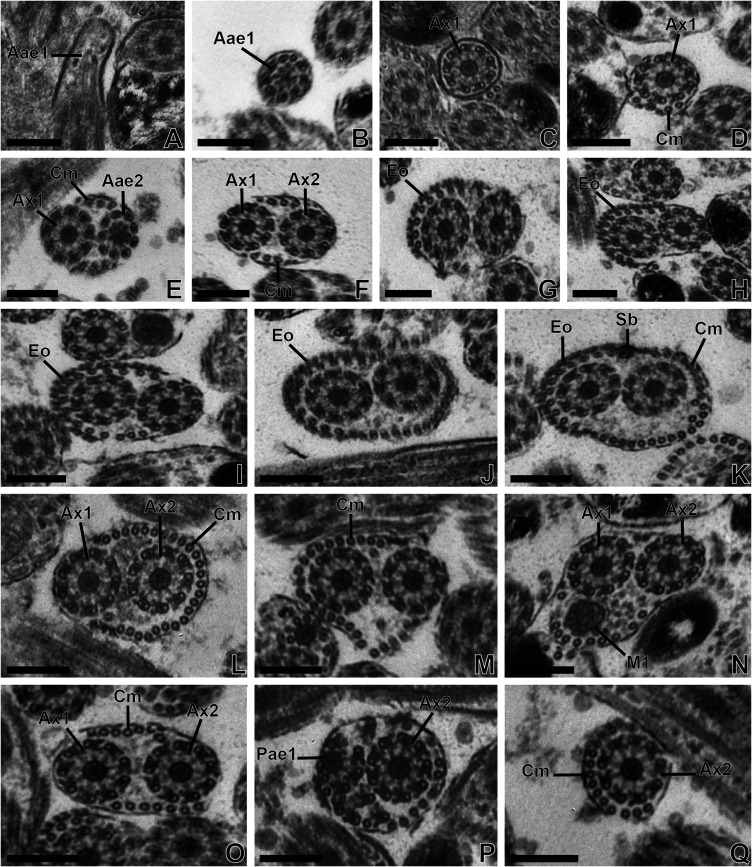
(A–Q) Transmission electron micrographs of regions I, II and III of the mature spermatozoon of *Tergestia laticollis.* Scale bars = 0.2 μm. (A–F) Sections in the anterior extremity of the spermatozoon. (A–B). Longitudinal (A) and cross-sections (B) in the anterior extremity of the spermatozoon with only the centriole of axoneme 1 (Aae1). (C) Cross-section in the anterior extremity of the spermatozoon with only one axoneme (A × 1). (D) Cross-section in the anterior extremity of the spermatozoon with axoneme 1 and three cortical microtubules. (E–F) Cross-section in region I of the spermatozoon with axoneme 1 and the centriole of axoneme 2 (Aae2) (E), then the two axonemes (A × 1 and A × 2) (F). (G–K) Cross-sections in region II of the mature spermatozoon showing the two axonemes, an increasing number of cortical microtubules up to 36 in (K), and the external ornamentation of the plasma membrane (Eo) and spine-like body (Sb) (K). (L–Q) Cross-sections in region III of the mature spermatozoon showing only two axonemes and a row of 26 cortical microtubules (L), 25 (M), the appearance of the first mitochondrion (M1) (N). Then, the disappearance of the first mitochondrion (O), disappearance of axoneme 1 (Pae1) and the disorganization of axoneme 1 (P). (Q) Cross-section in the extremity of region III showing only a row of 13 cortical microtubules and axoneme 2.

**Figure 4:**
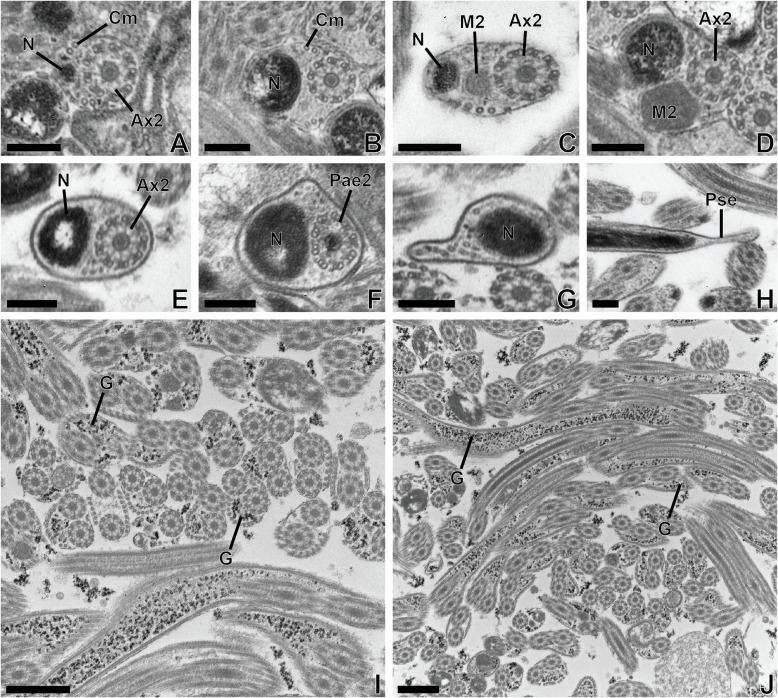
(A–H) Transmission electron micrographs of the posterior region (or region IV) of the mature spermatozoon of *Tergestia laticollis.* Scale bars = 0.2 μm. Cross-sections in region IV of mature spermatozoon of *Tergestia laticollis* showing the appearance of the nucleus (A–B), then the second mitochondrion (M2) (C). (D–E) Cross-sections showing progressively the disappearance firstly of the second mitochondrion and cortical microtubules. (F) Cross-section showing the disorganization of axoneme 2 (Pae2). (G) Cross-section in the posterior extremity of the spermatozoon exhibiting only the nucleus and a few granules of glycogen. (I–J). Transmission electron micrograph showing many sections of the mature spermatozoon of *Tergestia laticollis* with granules of glycogen (G) highlighted by the Thiéry method. Scale bars = 0.5 μm.

**Figure 5:**
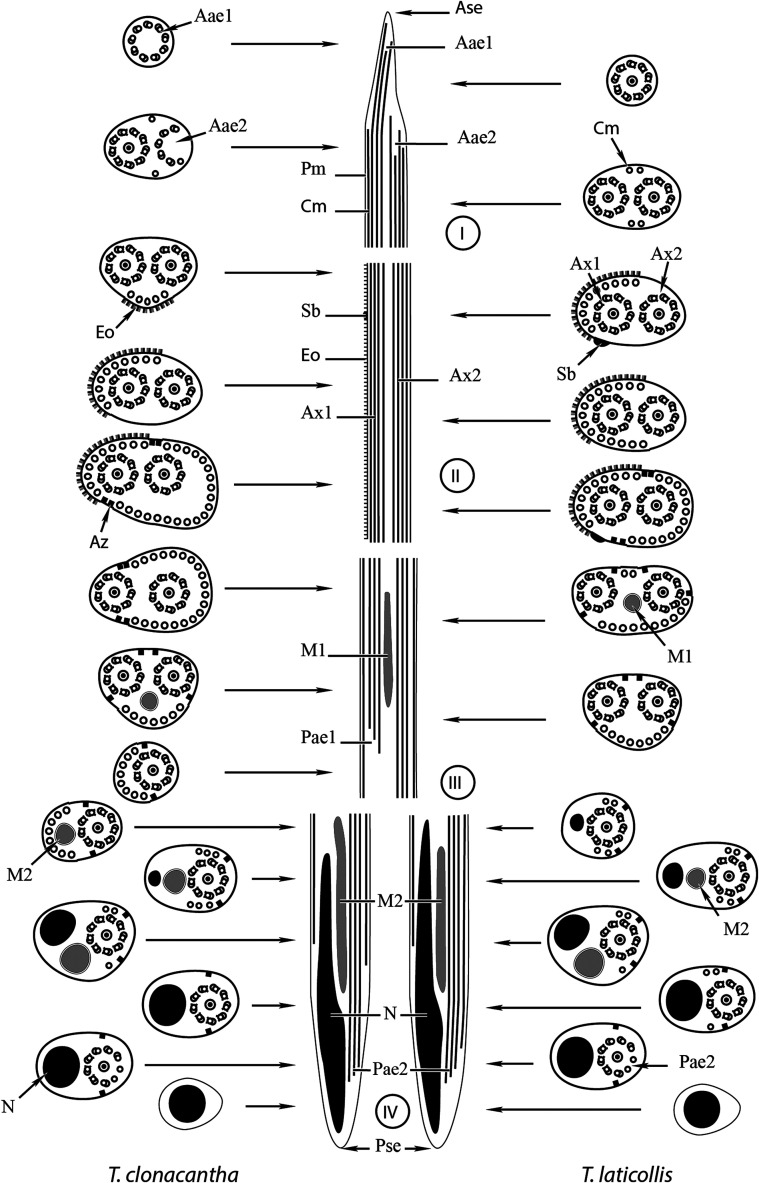
Schematic reconstruction of the mature spermatozoa of *Tergestia clonacantha* and *T. laticollis.* Aae1 = anterior extremity of the first axoneme, Aae2 = anterior extremity of the second axoneme, Ase = anterior spermatozoon extremity, A × 1 = first axoneme, A × 2 = second axoneme, Az = attachment zone, Cm = cortical microtubules, Eo = external ornamentation, M1 = first mitochondrion, M2 = second mitochondrion, N = nucleus, Pae1 = posterior extremity of the first axoneme, Pae2 = posterior extremity of the second axoneme, Pm = plasma membrane, Pse = posterior spermatozoon extremity, Sb = spine-like body.

*Region II* (Figs. 1D–1H, 3G–3K and 5II). In addition to the structures described in the posterior extremity of the region I of the spermatozoon, namely the two axonemes and cortical microtubules, we distinguish the presence of an external ornamentation of the plasma membrane (Figs 1D–1H and 3G–3K), spine-like bodies (Figs. 1H and 3K), and a significant increase in the number of cortical microtubules. This number increases from 5 microtubules in the anterior part to 36 in the posterior part of this region in *T. clonacantha* (Figs. 1D–1G) and from 6 to 36 in the case of *T. laticollis* (Figs. 3F–3K). In the two species, cross-sections of the spermatozoa at the posterior level of this region show the maximum number of cortical microtubules arranged in a continuous layer interrupted by attachment zones under the plasma membrane (Figs 1G and 3K). The external ornamentation of the plasma membrane is described on one side of the sections only in this region II (Figs. 1D–1H, 3G–3K and 5II).

*Region III* (Figs. 1I–1N, 3L–3Q and 5III). This region is characterized by the disappearance of spine-likes bodies and the external ornamentation of the plasma membrane, the presence of the first mitochondrion (Figs. 1I–1L and 3L–3N) and the disappearance of one of the axonemes in the posterior part of this region (Figs 1N and 3P–3Q). The number of cortical microtubules also decreases progressively, from 36 to 9 in *T. clonacantha* (Figs. 1M–1N) and from 36 to 13 in *T. laticollis* (Fig. 3Q).

*Region IV* (Figs. 2A–2E, 4A–4H, 5IV). This posterior region of the spermatozoa exhibits some differences between the two species.

In *T. clonacantha,* it is characterized by the appearance of the second mitochondrion in addition to the axoneme and cortical microtubules (Fig. 2A). The nucleus appears posterior to the anterior end of the mitochondrion (Fig. 2B). The second mitochondrion disappears at the posterior extremity of the spermatozoon (Fig. 2C), then the cortical microtubules, then the axoneme (Figs. 2D–2E). Thus, the cross-section of the posterior extremity of the spermatozoon shows the presence of the nucleus only (Fig. 2E).

In the case of *T. laticollis,* cross-sections in the anterior part of this region exhibit the appearance of the nucleus in addition to the second axoneme and cortical microtubules (Figs. 4A–4B), then the second mitochondrion (Fig. 4C). Cross-sections toward the posterior extremity show a progressive disappearance of cortical microtubules, then the second mitochondrion, and finally the axoneme (Figs. 4D–4F). The posterior extremity of the spermatozoon exhibits the presence of the nucleus only (Figs. 4G–4H).

Use of Thiéry’s method demonstrated the presence of glycogen granules in the mature spermatozoa of *Tergestia clonacantha* and *T. laticollis* (Figs. 2F–2G and 4I–4J).

## Discussion

The ultrastructure of the mature spermatozoon of *Tergestia clonacantha* and *T. laticollis* shows the general features described in the spermatozoon of most digeneans, namely: two axonemes with the 9 + “1” pattern of the Trepaxonemata, a nucleus, a mitochondrion or two, cortical microtubules, external ornamentation of the plasma membrane, spine-like bodies, and granules of glycogen. However, the mature spermatozoa of *T. clonacantha* and *T. laticollis* exhibit some peculiarities and give additional spermatological data for a better understanding of the relationships between the Gymnophalloidea and other digenean superfamilies. As is the case in many species of the superfamilies Gorgoderoidea, Lepocreadioidea, Opecoeloidea and Plagiorchioidea [7] with spermatozoon of type III or IV, we also found two mitochondria in the spermatozoa of *T. clonacantha* and *T. laticollis*. These are located in the posterior part of the mature spermatozoon in *T. clonacantha* and *T. laticollis,* in contrast to *T. acanthocephala* [13] which has the first mitochondrion in the anterior part of the spermatozoon. In addition, the mature spermatozoon of *T. acanthocephala* was described as similar to type IV proposed by Bakhoum et al. [7] but spermatozoa of *T. clonacantha* and *T. laticollis* (present study) exhibit type III. Thus, the assignation of type IV for spermatozoa of Gymnophalloidea in Bakhoum et al. [7] should be revised. This demonstrates the need for more studies in this superfamily to elucidate ultrastructural characteristics of the spermatozoa and their relationships. The ultrastructural model of the mature spermatozoon described in *T. clonacantha* and *T. laticollis* by the present study highlights some differences between them, mainly in the posterior region of the spermatozoa. The posterior extremity of the spermatozoon is characterized in the two species by the presence of the nucleus only, as in *T. acanthocephala* [13]. Nevertheless, in the case of *T. clonacantha*, before this extremity, we described successively the appearance of the second mitochondrion, then the nucleus, then the disappearance of cortical microtubules, then the extremity of the second axoneme, and finally the posterior extremity of the nucleus. In parallel and successively, in *T. laticollis,* the nucleus appears, then the second mitochondrion, then the end of cortical microtubules, then the posterior extremity of the second axoneme, and finally the posterior extremity of the nucleus.

Comparative ultrastructural studies of the mature spermatozoon have been performed in several species belonging to the same genus, within the Digenea. Overall, the authors found a similar general pattern. Table 1 shows that the same spermatozoon type (according to Bakhoum et al. [7]) has usually been found for species of the same genus: type III for *Holorchis micracanthum* [3] and *H. pycnoporus* [12]; type V for *Prosorhynchus aculeatus* and [18] *P. longisaccatus* [26]; type V for the Diplodiscidae *Diplodiscus amphichrus* [10] and *D. subclavatus* [5]; type V for *Fasciola gigantica* [22] and *F. hepatica* [21]; type II for *Lecithochirium microstomum* [23] and *L. musculus* [23]; type III for the Lepocreadiidae *Bianium arabicum* [30] and *B. plicitum* [30]; type IV for the Opisthorchiidae *Opisthorchis felineus* [32] and *O. viverrini* [13]; type IV for the Opecoelidae *Allopodocotyle pedicellata* [6] and *A. tunisiensis* [15], and type III for the Opecoelidae *Nicolla testiobliquum* [27] and *N. wisniewskii* [28]. Nevertheless, some differences have been observed between spermatozoa of species belonging to the same genus like the Notocotylidae *Notocotylus neyrai* [20] (undefined type) and *N. noyeri* [24] (type IV) concerning the location of the external ornamentation; the Opecoeloidea *Helicometra epinepheli* [29] (type III) and *Helicometra fasciata* [16] (undefined type) concerning especially the presence/absence of external ornamentation; and the Zoogonidae *Lecithostaphylus parexocoeti* [4] (undefined type) and *L. retroflexus* [14] (type IV) concerning the location of the external ornamentation.

**Table 1:** Comparative data on the ultrastructure of the mature spermatozoon in some digeneans of the same genus and their origins.

Families and species	Spermatozoon characters												Spermatozoon type	Reference	Host and host origin
	Principal characters								Secondary characters						
	TAx	Le	Eo	Eo + Cm	LEo	BCm	LMCm	M	Adm	Sb	Cob	Psc			
Aephnidiogenidae															
*Holorchis micracanthum*	9 + “1”	−	+	+	PostA	2	MedS	1	+	−	−	Ax	III	[3]	*Plectorhinchus mediterraneus* (Senegal)
*Holorchis pycnoporus*	9 + “1”	−	+	+	PostA	2	MedS	2	+	−	−	Ax	III	[12]	*Lithognathus mormyrus* (Tunisia)
Bucephalidae															
*Prosorhynchus aculeatus*	9 + “1”	+	+	+	AntA	2	AntS	1	−	+	−	Ax	V	[18]	*Conger conger* (Spain)
*Prosorhynchus longisaccatus*	9 + “1”	+	+	+	AntA	2	AntS	1	−	+	−	Ax	V	[26]	*Epinephelus maculatus* (New Caledonia)
Dicrocoeliidae															
*Dicrocoelium dendriticum*	9 + “1”	−	− ?	− ?	− ?	2	AntS	1	−	−?	−	Ax	?	[8]	Sheep (Unknown origin)
*Dicrocoelium hospes*	9 + “1”	−	+	+	PostA	2	AntS	2	−	+	−	N	IV	[1]	*Bos indicus* (Senegal)
Diplodiscidae															
*Diplodiscus amphichrus*	9 + “1”	+	+	+	AntA	2	AntS	1	−	+	−	N	V	[10]	*Hoplobatrachus rugulosus* (Thailand)
*Diplodiscus subclavatus*	9 + “1”	+	+	+	AntA	2	AntS	1	−	+	−	N	V	[5]	*Rana lessonae* (Belarus)
Fasciolidae															
*Fasciola gigantica*	9 + “1”	+	+	+	AntA	2	AntS	1	−	+	−	N	V	[22]	*Bos indicus* (Senegal)
*Fasciola hepatica*	9 + “1”	+	+	+	AntA	2	AntS	1?	−	+	−	N	V	[21]	*Bos taurus* (Spain)*, Rattus rattus* (France)
Fellodistomidae															
Tergestia acanthocephala	9 + “1”	−	+	+	PostA	2	AntS	2	−	+	−	N	IV	[13]	*Belone belone gracilis* (Tunisia)
*Tergestia clonacantha*	9 + “1”	−	+	+	PostA	2	MedS	2	−	+	−	N	III	Present study	*Hemirhamphus far* (New Caledonia)
*Tergestia laticollis*	9 + “1”	−	+	+	PostA	2	MedS	2	−	+	−	N	III	Present study	*Carangoides chrysophrys* (New Caledonia)
Hemiuridae															
*Lecithochirium microstomum*	9 + “1”	−	+	+	AntA	2	MedS	1	−	−	−	Ax	II	[23]	*Trichiurus lepturus* (Senegal)
*Lecithochirium musculus*	9 + “1”	−	+	+	AntA	2	MedS	1	−	−	−	Ax	II	[23]	*Anguilla anguilla* Corsica (France)
Lepocreadiidae															
*Bianium arabicum*	9 + “1”	+	+	+	PostA	2	MedS	2	+	+	−	N	III	[30]	*Lagocephalus sceleratus* (New Caledonia)
*Bianium plicitum*	9 + “1”	+	+	+	PostA	2	MedS	2	+	+	−	N	III	[30]	*Lagocephalus laevigatus* (Senegal)
Notocotylidae															
*Notocotylus neyrai*	9 + “1”	−	+	+	AntA	2	AntS	2	−	+	−	Ax	Undefined	[20]	*Microtus agrestis* (Spain)
*Notocotylus noyeri*	9 + “1”	−	+	+	PostA	2	AntS	2	−	+	−	Ax	IV	[24]	*Microtus arvalis* (Belarus)
Opecoelidae															
*Allopodocotyle pedicellata*	9 + “1”	−	+	+	PostA	2	AntS	2	+	+	−	Cm	IV	[6]	*Sparus aurata* (Tunisia)
*Allopodocotyle tunisiensis*	9 + “1”	−	+	+	PostA	2	AntS	2	+	+	−	Cm	IV	[15]	*Solea aegyptiaca* (Tunisia)
*Helicometra epinepheli*	9 + “1”	−	+	+	PostA	2	MedS	2	+	+	−	Cm	III	[29]	*Epinephelus fasciatus* (New Caledonia)
*Helicometra fasciata*	9 + “1”	−	−	−	−	2	MedS	1	+	−	−	Cm	Undefined	[16]	*Labrus merula* (France)
*Nicolla testiobliquum*	9 + “1”	−	+	+	PostA	2	MedS	2	−	+	−	Cm	III	[27]	*Salmo trutta* (France)
*Nicolla wisniewskii*	9 + “1”	−	+	+	PostA	2	MedS	1?	−	+	−	Cm	III	[28]	*Salmo trutta* (France)
Opisthorchiidae															
*Opisthorchis felineus*	9 + “1”	−	+	+	PostA	2	AntS	2	−	−	−	N	IV	[32]	*Mesocricetus auratus* (Unknown origin)
*Opisthorchis viverrini*	9 + “1”	−	+	+	PostA	2	AntS	2	−	+	−	Ax	IV	[19]	*Mesocricetus auratus* (Thailand)
Zoogonidae															
*Lecithostaphylus parexocoeti*	9 + “1”	−	+	+	AntA	2	AntS	2	−	+	−	N	Undefined	[4]	*Cheilopogon pinnatibarbatus* (Senegal)
*Lecithostaphylus retroflexus*	9 + “1”	−	+	+	PostA	2	AntS	2	−	+	−	N	IV	[14]	*Belone belone gracilis* (Tunisia)

Adm, anterior electron-dense material; AntA, anterior part of the anterior region; AntS, anterior region of the spermatozoon; Ax, axoneme; BCm, number of bundles of cortical microtubules; Cm, cortical microtubules; Cob, cytoplasmic ornamented buttons; Eo, external ornamentation of the plasma membrane; Eo + Cm, association of external ornamentation with cortical microtubules; Le, lateral expansion; LEo, location of the external ornamentation; LMCm, location of maximum number of cortical microtubules; M, number of mitochondria; MedS, median part of the spermatozoon; N, nucleus; PostA, posterior to the anterior region; Psc, posterior spermatozoon character; Sb, spine-like bodies; TAx, type of axoneme; +/−, presence/absence of considered character; ?, doubtful or unknown data.

The data shown in Table 1 indicate that that the general model of the spermatozoon is usually the same when the species belong to the same genus but can, in some case, differ according to one ultrastructural character.

## Conflict of interest

The Editor-in-Chief of Parasite is one of the authors of this manuscript. COPE (Committee on Publication Ethics, http://publicationethics.org), to which Parasite adheres, advises special treatment in these cases. In this case, the peer-review process was handled by an Invited Editor, Jérôme Depaquit.

## References

[1] Agostini S, Miquel J, Ndiaye PI, Marchand B. 2005. *Dicrocoelium hospes* Looss, 1907 (Digenea, Dicrocoeliidae): spermiogenesis, mature spermatozoon and ultrastructural comparative study. Parasitology Research, 96, 38–48. 15772868 10.1007/s00436-005-1318-6

[2] Bâ CT, Marchand B. 1995. Spermiogenesis, Spermatozoa and phyletics affinities in the Cestoda, in Advances in Spermatozoal Phylogeny and Taxonomy, Jamieson BGM, Ausio J, Justine J-L, Editors. Mémoires du Muséum National d’Histoire Naturelle: Paris, 166. p. 87–95.

[3] Bâ CT, Ndiaye PI, Dione A, Quilichini Y, Marchand B. 2011. Ultrastructure of the spermatozoon of *Holorchis micracanthum* (Digenea: Lepocreadiidae), an intestinal parasite of *Plectorhinchus mediterraneus* (Pisces, Teleostei) in Senegal. Parasitology Research, 109, 1099–1106. 21445614 10.1007/s00436-011-2352-1

[4] Bâ A, Bakhoum AJS, Bâ CT, Bray RA, Marchand B, Ndiaye PI, Quilichini Y. 2020. Ultrastructure of the spermatozoon of *Lecithostaphylus parexocoeti* (Digenea, Microphalloidea, Zoogonidae) parasite of the flying fish *Cheilopogon pinnatibarbatus* (Teleostei, Exocoetidae) of Senegal and their implication on the phylogenetic relationships in Microphalloidea. Zoomorphology, 139, 319–326.

[5] Bakhoum AJS, Torres J, Shimalov VV, Bâ CT, Miquel J. 2011. Spermiogenesis and spermatozoon ultrastructure of *Diplodiscus subclavatus* (Pallas, 1760) (Paramphistomoidea, Diplodiscidae), an intestinal fluke of the pool frog *Rana lessonae* (Amphibia, Anura). Parasitology International, 60, 64–74. 20974288 10.1016/j.parint.2010.10.006

[6] Bakhoum AJS, Kacem H, Neifar L, Miquel J. 2017. The Opecoelidae sperm model and its contribution to phylogeny: spermatozoon ultrastructural particularities of *Allopodocotyle pedicellata* (Plagioporinae, Digenea, Platyhelminthes). Zoologischer Anzeiger, 266, 28–34.

[7] Bakhoum AJS, Miquel J, Ndiaye PI, Justine J-L, Falchi A, Bâ CT, Marchand B, Quilichini Y. 2017. Advances in spermatological characters in the Digenea: review and proposal of spermatozoa models and their phylogenetic importance. Advances in Parasitology, 98, 111–165. 28942768 10.1016/bs.apar.2017.04.001

[8] Cifrian B, Garcia-Corrales P, Martinez-Alos S. 1993. Ultrastructural study of the spermatogenesis and mature spermatozoa of *Dicrocoelium dendriticum* (Plathelminthes, Digenea). Parasitology Research, 79, 204–212. 8493244 10.1007/BF00931894

[9] Cribb TH, Bray RA, Littlewood DTJ, Pichelin SP, Herniou EA. 2001. The Digenean, in Interrelationships of the Platyhelminthes, Littlewood DTJ, Bray RA, Editors. Taylor and Francis: London, UK. p. 168–185.

[10] Diagne PM, Ribas A, Poonlaphdecha S, Miquel J. 2020. Sperm characteristics in the digenean *Diplodiscus amphichrus* (Paramphistomoidea, Diplodiscidae), a parasite of the Chinese edible frog *Hoplobatrachus rugulosus*. Zoomorphology, 139, 309–317.

[11] Justine J-L. 1998. Spermatozoa as phylogenetic characters for the Eucestoda. Journal of Parasitology, 84, 385–408. 9576517

[12] Kacem H, Miquel J. 2020. Spermatological characters in the Lepocreadioidea, with first data on *Holorchis pycnoporus* (Aephnidiogenidae), a parasite of the striped seabream *Lithognathus mormyrus* (Sparidae) from the Gulf of Gabes (Tunisia). Tissue & Cell, 67, 101409. 32835942 10.1016/j.tice.2020.101409

[13] Kacem H, Ndiaye PI, Neifar L, Torres J, Miquel J. 2015. Ultrastructure of the spermatozoon of the digenean *Tergestia acanthocephala* (Stossich, 1887) (Gymnophalloidea: Fellodistomidae): an intestinal parasite of *Belone belone gracilis* (Pisces: Teleostei). Tissue & Cell, 47, 235–241. 25796547 10.1016/j.tice.2015.01.008

[14] Kacem H, Ndiaye PI, Neifar L, Torres J, Miquel J. 2015. Spermatological characters of the digenean *Lecithostaphylus retroflexus* (Molin, 1859) (Microphalloidea: Zoogonidae), a parasite of the teleost fish *Belone belone gracilis*. Tissue & Cell, 47, 431–437. 26025421 10.1016/j.tice.2015.05.003

[15] Kacem H, Diagne PM, Miquel J. 2019. Ultrastructural organisation of the spermatozoon of *Allopodocotyle tunisiensis* Derbel and Neifar, 2009 (Digenea, Opecoelidae), an intestinal parasite of *Solea aegyptiaca* Chabanaud, 1927 (Teleostei, Soleidae). Tissue & Cell, 57, 1–7. 30947958 10.1016/j.tice.2019.01.008

[16] Levron C, Ternengo S, Marchand B. 2003. Ultrastructure of spermiogenesis and the spermatozoon of *Helicometra fasciata* (Digenea, Opecoelidae), a parasite of *Labrus merula* (Pisces, Teleostei). Acta Parasitologica, 48, 255–264.

[17] Levron C, Miquel J, Oros M, Scholz T. 2010. Spermatozoa of tapeworms (Platyhelminthes, Eucestoda): advances in ultrastructural and phylogenetic studies. Biological Reviews, 85, 523–543. 20015312 10.1111/j.1469-185X.2009.00114.x

[18] Miquel J, Delgado E, Sarra L, Torres J. 2017. Sperm characters of the digenean *Prosorhynchus aculeatus* Odhner, 1905 (Bucephalidae), a parasite of the marine fish *Conger conger* (Linnaeus, 1758) (Congridae). Zoomorphology, 136, 299–305.

[19] Miquel J, Świderski Z, Sripa B, Ribas A. 2017. Ultrastructural characters of the spermatozoon of the liver fluke *Opisthorchis viverrini* (Poirier, 1886) (Opisthorchiidae). Parasitology Research, 116, 2499–2506. 28725936 10.1007/s00436-017-5559-y

[20] Ndiaye PI, Miquel J, Feliu C, Marchand B. 2003. Ultrastructure of spermiogenesis and spermatozoa of *Notocotylus neyrai* González Castro, 1945 (Digenea, Notocotylidae), intestinal parasite of *Microtus agrestis* (Rodentia: Arvicolidae) in Spain. Invertebrate Reproduction and Development, 43, 105–115.

[21] Ndiaye PI, Miquel J, Fons R, Marchand B. 2003. Spermiogenesis and sperm ultrastructure of the liver fluke *Fasciola hepatica* L., 1758 (Digenea, Fasciolidae): transmission and scanning electron microscopy, and tubulin immunocytochemistry. Acta Parasitologica, 48, 182–194.

[22] Ndiaye PI, Miquel J, Bâ CT, Marchand B. 2004. Spermiogenesis and ultrastructure of the spermatozoon of the liver fluke *Fasciola gigantica* cobbold, 1856 (Digenea: Fasciolidae), a parasite of cattle in Senegal. Journal of Parasitology, 90, 30–40. 10.1645/GE-3171 15040664

[23] Ndiaye PI, Quilichini Y, Sène A, Tkach VV, Bâ CT, Marchand B. 2014. Ultrastructural characters of the spermatozoa in digeneans of the genus *Lecithochirium* Lühe, 1901 (Digenea, Hemiuridae), parasites of fishes: comparative study of *L. microstomum* and *L. musculus*. Parasite, 21, 49. 25275216 10.1051/parasite/2014050 PMC4178227

[24] Ndiaye PI, Torres J, Eira C, Shimalov VV, Miquel J. 2015. Ultrastructure of the spermatozoon of the trematode *Notocotylus noyeri* (Digenea: Notocotylidae), a parasite of *Microtus arvalis* (Rodentia: Cricetidae). Folia Parasitologica, 62, 001. 10.14411/fp.2015.001 25960545

[25] Ndiaye PI, Miquel J, Marchand B. 2016. Systématique et phylogénie de Plathelminthes parasites, Trématodes et Cestodes: Apports des études ultrastructurales de la reproduction. Saarbrücken, Allemagne: Presses Académiques Francophones. 229 p.

[26] Ndiaye PI, Marchand B, Bâ CT, Justine J, Bray RA, Quilichini Y. 2018. Ultrastructure of mature spermatozoa of three Bucephalidae (*Prosorhynchus longisaccatus*, *Rhipidocotyle khalili* and *Bucephalus margaritae*) and phylogenetic implications. Parasite, 15, 65. 10.1051/parasite/2018065 PMC6284405 30526820

[27] Quilichini Y, Foata J, Marchand B. 2007. Ultrastructural study of the spermatozoon of *Nicolla testiobliquum* (Digenea, Opecoelidae) parasite of brown trout *Salmo trutta* (Pisces, Teleostei). Parasitology Research, 101, 1295–1301. 17628831 10.1007/s00436-007-0636-2

[28] Quilichini Y, Foata J, Orsini A, Marchand B. 2007. Spermiogenesis and spermatozoon ultrastructure of *Nicolla wisniewskii* (Digenea: Opecoelidae), an intestinal parasite of brown trout *Salmo trutta* (Pisces: Teleostei). Journal of Parasitology, 93, 469–478. 10.1645/GE-1085R.1 17626336

[29] Quilichini Y, Foata J, Justine JL, Bray RA, Marchand B. 2011. Sperm ultrastructure of *Helicometra epinepheli* (Platyhelminthes, Digenea, Opecoelidae), parasite of *Epinephelus fasciatus* (Pisces, Teleostei). Histology and Histopathology, 26, 1019–1028. 21692034 10.14670/HH-26.1019

[30] Quilichini Y, Ndiaye PI, Sène A, Justine J-L, Bray RA, Tkach VV, Bâ CT, Marchand B. 2015. Ultrastructural characters of the spermatozoa in digeneans of the genus *Bianium* Stunkard, 1930 (Digenea, Lepocreadiidae) parasites of fishes: a comparative study of *Bianium plicitum* and *Bianium arabicum*. Parasitology Research, 114, 3747–3757. 26220557 10.1007/s00436-015-4604-y

[31] Thiéry JP. 1967. Mise en évidence des polysaccharides sur coupes fines en microscopie électronique. Journal de Microscopie, 6, 987–1018.

[32] Zhukova MV, Mordvinov VA, Kiseleva E. 2014. Ultrastructure of spermatozoa in the seminal receptacle of the liver fluke *Opisthorchis felineus* (Rivolta, 1884). Parasitology Research, 113, 1093–1101. 24452915 10.1007/s00436-013-3746-z

